# Photonic integrated chip enabling orbital angular momentum multiplexing for quantum communication

**DOI:** 10.1515/nanoph-2021-0500

**Published:** 2021-11-30

**Authors:** Mujtaba Zahidy, Yaoxin Liu, Daniele Cozzolino, Yunhong Ding, Toshio Morioka, Leif K. Oxenløwe, Davide Bacco

**Affiliations:** Center for Silicon Photonics for Optical Communications (SPOC), Department of Photonics Engineering, Technical University of Denmark, Kgs. Lyngby, Denmark

**Keywords:** orbital angular momentum, quantum communication, quantum key distribution, silicon photonics

## Abstract

Light carrying orbital angular momentum constitutes an important resource for both classical and quantum information technologies. Its inherently unbounded nature can be exploited to generate high-dimensional quantum states or for channel multiplexing in classical and quantum communication in order to significantly boost the data capacity and the secret key rate, respectively. While the big potentials of light owning orbital angular momentum have been widely ascertained, its technological deployment is still limited by the difficulties deriving from the fabrication of integrated and scalable photonic devices able to generate and manipulate it. Here, we present a photonic integrated chip able to excite orbital angular momentum modes in an 800 m long ring-core fiber, allowing us to perform parallel quantum key distribution using two and three different modes simultaneously. The experiment sets the first steps towards quantum orbital angular momentum division multiplexing enabled by a compact and light-weight silicon chip, and further pushes the development of integrated scalable devices supporting orbital angular momentum modes.

## 1 Introduction

Since 1992, when L. Allen and colleagues discovered that Laguerre–Gaussian beams have a well-defined orbital angular momentum (OAM) [1], an enormous amount of research has been carried out to better understand and manipulate light owning a nonzero OAM [2]. Optical beams possessing a well-defined OAM are characterized by the azimuthal phase dependence e^i*ℓφ*^, where *ℓ*ℏ is the OAM carried by each photon, with ℏ being the reduced Planck constant; *ℓ* is the topological charge, an integer that specifies the OAM value, and *φ* is the azimuthal angle. Such helical phase twists along its propagation axis and determines the cancellation of the light beam at the axis itself, thus resulting in a “doughnut” intensity profile. It is thanks to these special features, i.e., intensity and phase structures, that light owning an OAM has been applied in many fields of optics, e.g., optical trapping [3, 4] or quantum information [5, 6]. In the last decade, OAM has been largely investigated in the field of fiber-based optical communication, both classical and quantum, achieving unprecedented results that have forecast its exploitation to real-case scenarios [7–9]. It has shown great potentials in communication systems due to the unbounded nature of the topological charge *ℓ* and the inherent orthogonality between optical modes or quantum states. Indeed, these characteristics are exceptional resources for optical mode multiplexing and high-dimensional quantum communication: the former aims to overcome the channel capacity crunch in classical communication systems [10, 11] or to boost photon information efficiency in the quantum ones [8]; the latter, uses quantum states encoded in a large Hilbert space, i.e., high-dimensional quantum states, as they can tolerate higher noise thresholds, thus they are useful for communications over noisy channels or in extreme conditions, such as photon starving or detector saturation regimes [12, 13]. Nonetheless, despite the results achieved hitherto, applications of OAM beyond proof-of-principle experiments require the development of integrated devices able to generate, transmit, and manipulate such a degree of freedom. On-chip generation of OAM modes both for classical and quantum applications, have been demonstrated using star couplers [14], microring resonators [15], and controlled phase arrays [16]. Also, the transmission of OAM modes through a silica chip has been investigated [17], as well as the combination of an integrated optical emitter and a ring-core fiber for classical communication [18].

**Figure 1:**
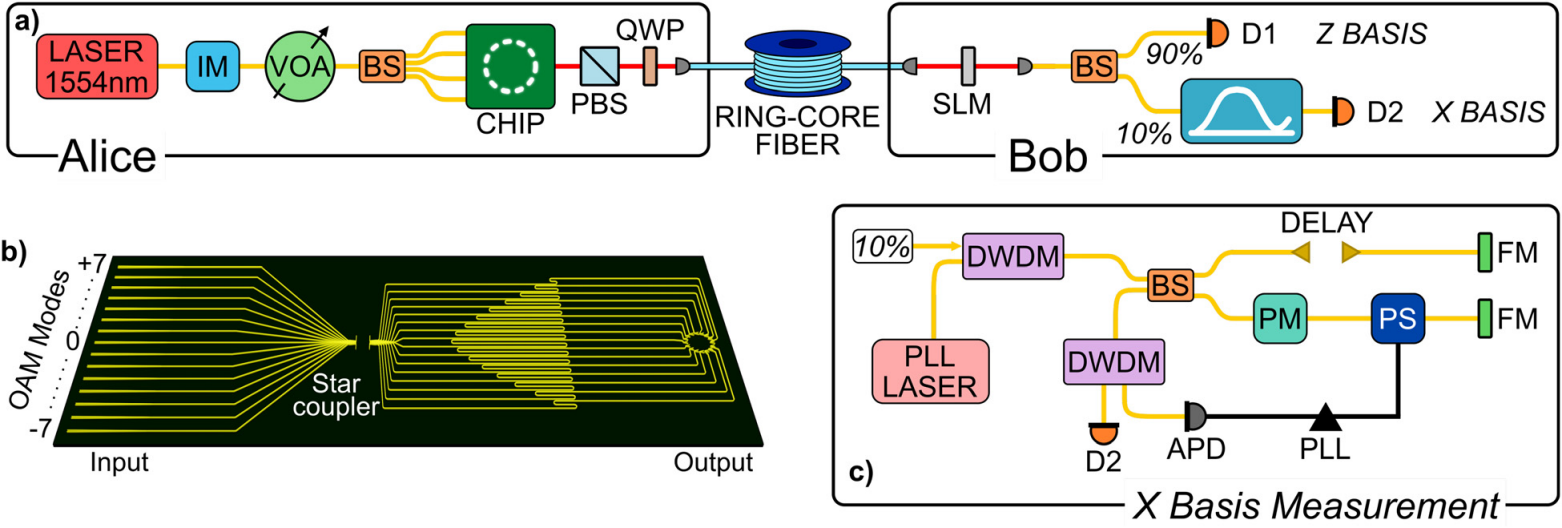
Realization of OAM multiplexing, time-bin encoding quantum key distribution. (a) Experimental setup. IM: intensity modulator; VOA: variable optical attenuator; BS: beam-splitter; PBS: polarization beam-splitter; QWP: quarter-wave plate; SLM: spatial light modulator; D*i*: *i*th detector. (b) Schematic of the photonic integrated device. (c) *X* basis measurement setup. PLL: phase-locked loop; DWDM: dense wavelength-division-multiplexing; PM: phase modulator; PS: phase shifter; FM: Faraday mirror; APD: avalanche photodiode; PLL: phase-locked loop.

In this work, we exploit a photonic integrated emitter based on the star-coupler technology to seed an 800 m long ring-core fiber with a three-times OAM multiplexed quantum key distribution (QKD) protocol, using time-bin encoded states. We believe our work further closes the gap between proof-of-principle experiments and concrete deployment of OAM-based technologies, thus foreseeing them as a near-future reach (Figure 1).

## 2 Excitation of the orbital angular momentum fiber modes

**Figure 2:**
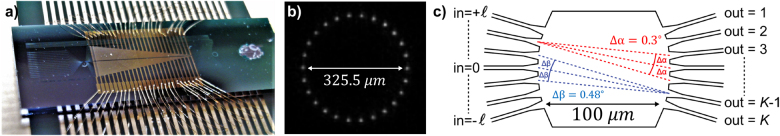
Compact and scalable design of photonic integrated chips. (a) Picture shows the photonic integrated device used in the experiment. (b) Infrared image of the grating couplers output. The 26 output are arranged on a ring of 325.5 μm. (c) Schematic of the star-coupler structure.

The main goal of our experiment is to spatially multiplex and transmit time-bin encoded QKD signals using the OAM degree of freedom. In this regard, a fundamental role is played by our integrated device, which is a silicon-on-insulator chip that, given an input, outputs a ring of Gaussian spots with a well-defined relative phase [19]. The OAM chip function is to imitate the radial phase pattern and characteristics of a definite OAM mode. The overall field will excite the intended OAM mode in the fiber upon coupling with a coupling efficiency which corresponds to the overlap of the output field with the OAM mode. Figure 2(a) and (b) shows the fabricated device and its output. The chip consists of three main parts: the input grating couplers, a star coupler and a ring of output couplers, and it supports the multiplexing to OAM modes within *ℓ* = −7 to *ℓ* = +7. There are 15 input grating couplers with a 500 μm adiabatic taper, whose spacing is 127 μm that is compatible with commercially available fiber arrays. Each input can be labeled with integers ranging from −*ℓ* to +*ℓ*.

The star coupler [14], schematically shown in Figure 2(c), is an optical element that distributes an incoming signal into *K* output waveguides with phase differences of Δ*φ* = 2*πℓ*/*K* for neighboring output waveguides, so that the total phase difference across all output waveguides is 2*πℓ*, where *ℓ* depends on the chosen input waveguide. In our case, *ℓ* lies within −7 and +7 and *K* = 26 arranged on ring with 325.5 μm diameter. The input and output waveguides are spread at equidistant angles Δ*α* = 0.3° and Δ*β* = 0.48°, respectively. The 26 output ports are connected to a ring of 26 grating couplers oriented in the same direction allowing coupling of the light at 15° angle. Figure 2(b) shows an image of the 26 grating couplers output. The optical path length of the 26 waveguides between the star coupler and the ring of output couplers are identical, nonetheless, each waveguide is supported with a thermal heater that allows for phase compensation to account for fabrication tolerances. The chip has approximately 22 dB of losses, stemming from input coupling loss, waveguides and other components losses, and output grating couplers losses.

A specific OAM fiber mode ±*ℓ* can be excited by injecting light into the respective ±*ℓ* input, so that the output ring of Gaussian spots is generated, whose total phase difference is 2*πℓ*. The injection of light into more inputs simultaneously enables the excitation of different OAM fiber modes, thus realizing the OAM mode multiplexing.

## 3 Experimental setup

The QKD protocol we implemented and multiplexed using OAM modes is the three-state time-bin protocol, where we have used the 1-decoy technique and carried out the finite key analysis [20]. The experimental setup we realized is shown in Figure 1. A continuous wave laser at 1554.13 nm, channel 29 of the International Telecommunication Union-Telecommunication Standardization Sector (ITU-T), is carved to form a train of pulses at a repetition rate of 595 MHz, which are subsequently attenuated to reach the single photon level and form the time-bin qubits exploited in the experiment. The carving procedure is performed by two cascaded intensity modulators, shown as one in Figure 1, controlled by a field programmable gate array (FPGA), which generates electrical signals according to a pseudo-random binary sequence of length *l* = 2^12^ − 1.

**Figure 3:**
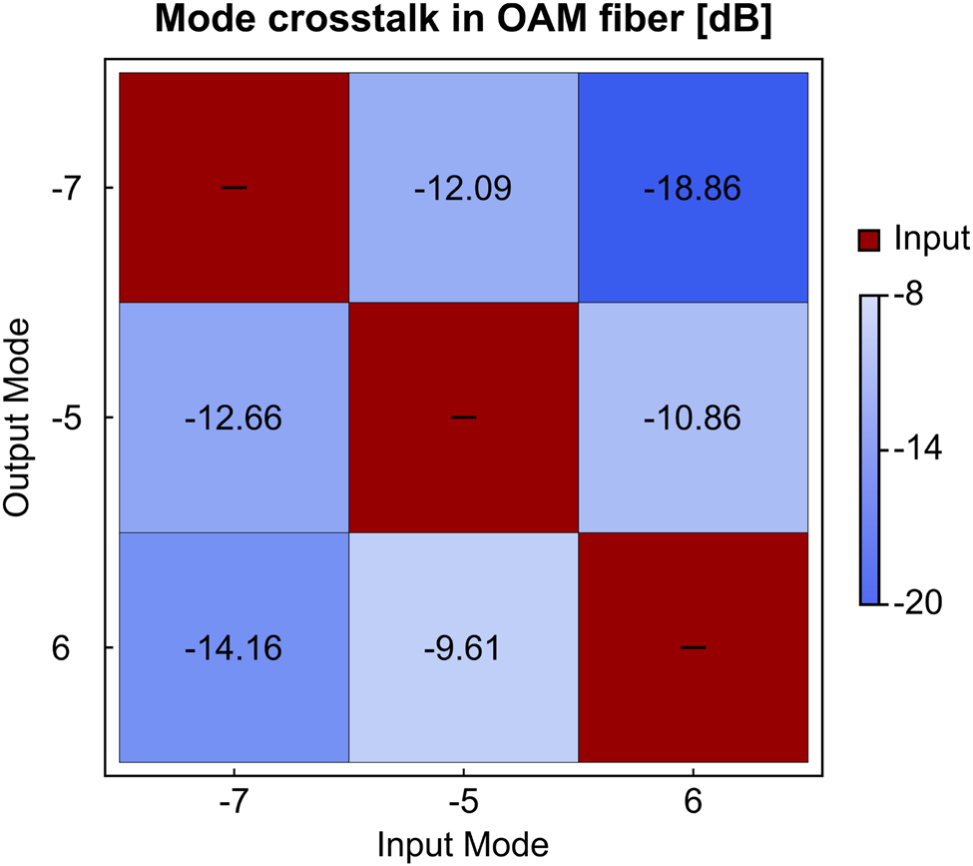
Mode crosstalk matrix in OAM fiber. On the *x* axis, we report the inputs mode (−7, −5 and 6). On the *y* axis we report the output mode (−7, −5 and 6). The measurement has been normalized per each mode.

It should be noted that to block the loopholes and guarantee the security of the real QKD implementation, quantum states should be phase randomized and the pseudo-random sequence should be replaced with a quantum random number generator [21]. Furthermore, in addition to providing the electrical signals driving the intensity modulators, the FPGA generates an electrical signal, of width 1.68 ns and a repetition rate of 145.358 KHz, which is used for clock synchronization. However, due to source and receiver physically situated remote, an optical synchronization scheme was implemented. Following the cascaded intensity modulators, the quantum state signal is then split by using a 1 by 4 beam-splitter and fed to the integrated chip. Since the integrated chip is polarization sensitive, polarization controllers are used to maximize coupling of individual signals into the chip. Finally, the chip output is collimated, and its polarization is transformed from linear to circular by the combination of a polarization beam-splitter and a quarter-wave plate. After that, the chip output is coupled into the ring-core fiber, corresponding to the quantum channel, exciting the desired OAM modes, each of them generating an independent key. For the purpose of this demonstration, we choose 2(3) modes, −7 and −5 (−7, −5 and +6) which showed a crosstalk as low as ≈−12 dB (≈−18 dB), see Figure 3. The OAM modes crosstalk stems from misalignment of the chip output to the OAM fiber, mismatch of the chip outputs relative phase induced by the heaters, as well as bends and twists of the fiber itself. Since the group velocity of each mode in the OAM carrying fiber is different for each of them, the mode crosstalk can be directly measured and optimized with a time-of-flight measurement [8]. A further analysis of crosstalk was performed via power measurement. Exciting only one mode at a time, a spatial light modulator (SLM) is used to demultiplex the output with various modes. The power coupled to the single mode fiber is then compared with the excited mode and crosstalk is measured. In our case, a further optimization has been possible by performing multiple runs of phase optimization for the chip output [22]. At the receiver, Bob, OAM modes demultiplexing is performed by means of an SLM and one mode at a time. The Gaussian beam obtained from the SLM conversion is then coupled to a single mode fiber, which also filters out the unwanted modes residue. A combination of ≈40 meter long patch cord fibers connects the signal to the SNSPDs where the same fiber is used to share the optical clock synchronization signal. Three DWDMs are used to multiplex (one) the clock signal, Ch33 (ITU-T), and demultiplex (two) the quantum signal from the optical clock distribution. Following the DWDMs, a 90:10 beam-splitter marks the beginning of the passive measurement stage. The 90% output, corresponding to the *Z* basis, is detected directly with a superconducting nano-wire single photon detector (SNSPD), while the 10% output, corresponding to the *X* basis, is redirected to an unbalanced Michelson interferometer with 800 ps time delay in one arm with respect to the other one, see Figure 1(c). The fiber-based Michelson interferometer consists of two Faraday rotator mirrors, a polarization controller, adjustable delay-line, and a piezoelectric phase shifter. To compensate for the relative phase drift of the interferometer arms, a phase lock loop is implemented [23]. A monitor laser (PLL laser) is mixed with the 10% part of the signal via a dense wavelength-division-multiplexing (DWDM) device and sent to the interferometer. At the interferometer output, a second DWDM separates the QKD signal and the PLL laser. By monitoring the PLL laser power with an avalanche photodiode it is then possible to actuate the phase shifter inside the interferometer and stabilize the interference fringes. To maximize the efficiency and the key-rate, SNSPDs were used with 83% efficiency, ≈50 counts per second of dark counts, and 33 ps of dead-time. Detection events and their time of arrival are registered by a time to digital converter with temporal resolution of 1 ps.

## 4 Results

One key parameter for QKD protocols is the quantum bit error rate (QBER). In particular, minimizing mode crosstalk is crucial for having low error rate in the QKD experiment. In this way, indeed, the error reconciliation part has less impact on the final secret key rate. In Figure 3, we report the results of the mode crosstalk measurements after 6 rounds of phase optimization followed by the alignment of the chip output to the OAM fiber. We then proceed to the implementation of the QKD experiment by sending all the modes at the same time and by demultiplexing one mode at a time. The channel loss is measured around 1 dB for 800 m of ring-core fiber, however, the signal and key rate suffered from an average 15 dB of coupling losses after the OAM-to-Gaussian mode conversion, and 9.15 dB of extra loss due to the optical synchronization scheme implemented.

**Figure 4:**
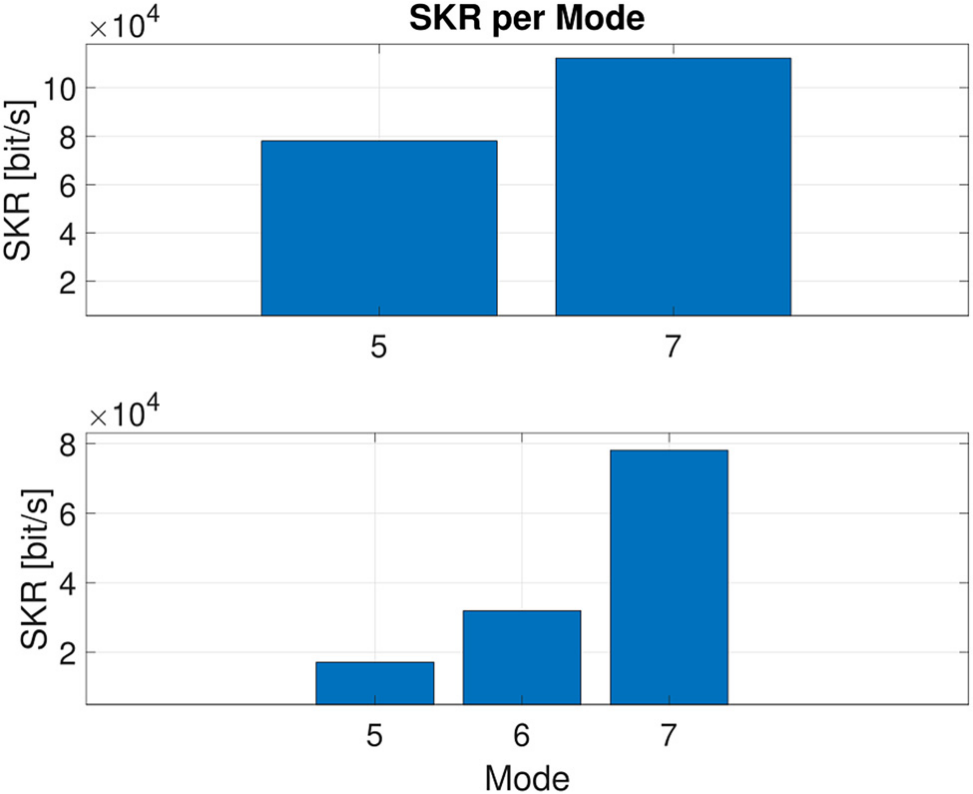
Secret key rate experimentally achieved for the 2-mode (top) and 3-mode (bottom) multiplexing.

**Figure 5:**
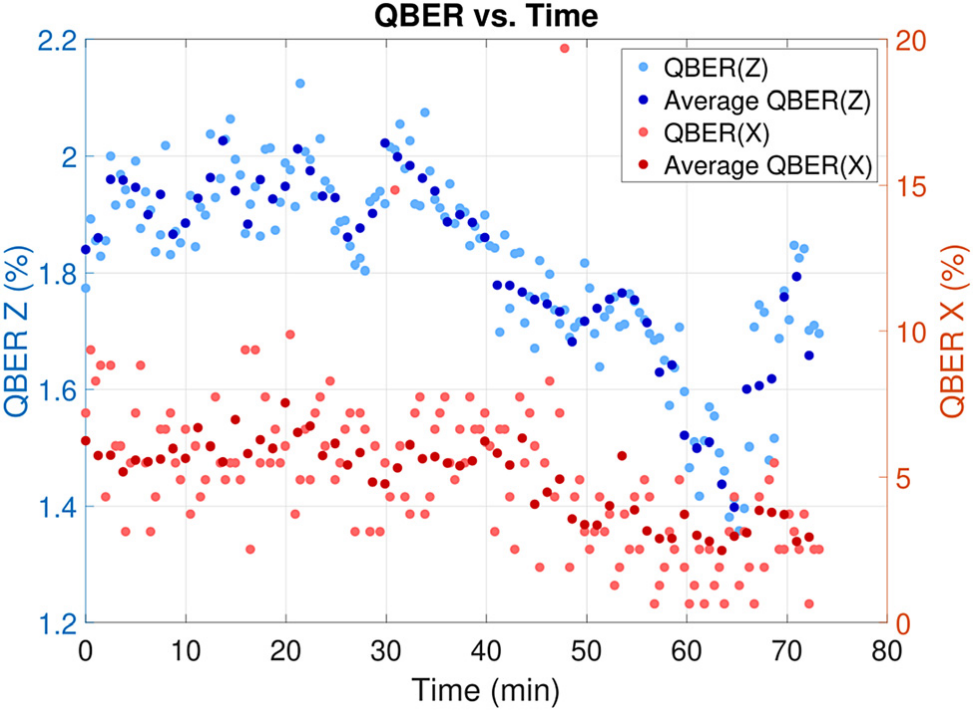
Stability of the QKD system. The graph represents variation of QBER measured in the *X* and *Z* basis for a duration of approximately 75 min. The averaging points represent average QBER obtained every 75 s.

Since the coupling losses of the demultiplexd light as well as the achievable QBERs were different for each mode due to the different amount of crosstalk, we estimated the optimal mean photon number in each case by using ad-hoc software simulation. These values are reported in Tables 1 and 2 for the multiplexing of 2 and 3 QKD signals, respectively. The QBER also suffered from background noise due to the optical synchronization scheme, which added ≈4 kcounts/s in the *Z* basis and ≈200 kcounts/s in the *X* basis. The secret key rate values obtained in each mode are shown in Figure 4. Each point has been measured for 5 min. In addition, in order to test the overall stability of the entire system, we decided to acquire a long measurement of the multiplexing quantum system. In Figure 5, the long-term stability of key-generation basis QBER, *Q*_*Z*_, and the security check, *Q*_*X*_, are presented. Data acquisition during a course of 75 min, while monitoring mode 7 with mean photon number set to *μ* = 0.24, shows a QBER of less than 2% in the *Z* basis and 6% in the *X* basis. It should be noted that the system is stable for more than an hour and the overall QBER improves during the measurement process. From previous experiences with a similar setup, we can confirm that this effect is due to the phase drift of the heaters in the chip and to the intrinsic stabilization of our measurement apparatus.

**Table 1:** Experimental mean photon numbers for the 2-mode multiplexing demonstration as well as QBERs and SKR attained for each mode.

	Mode 7	Mode 5
*μ*_1_	0.26	0.36
*μ*_2_	0.13	0.13
${\text{QBER}}_{Z_{\mu_{1}}}$	2.15	1.35
${\text{QBER}}_{Z_{\mu_{2}}}$	2.13	2.00
${\text{QBER}}_{X_{\mu_{1}}}$	4.23	3.53
${\text{QBER}}_{X_{\mu_{2}}}$	4.08	4.12

**Table 2:** Experimental mean photon numbers for the 3-mode multiplexing demonstration as well as QBERs and SKR attained for each mode.

	Mode 7	Mode 6	Mode 5
*μ*_1_	0.28	0.41	0.46
*μ*_2_	0.18	0.28	0.305
${\text{QBER}}_{Z_{\mu_{1}}}$	1.81	4.47	2.30
${\text{QBER}}_{Z_{\mu_{2}}}$	1.92	4.28	2.09
${\text{QBER}}_{X_{\mu_{1}}}$	6.24	6.75	5.89
${\text{QBER}}_{X_{\mu_{2}}}$	6.02	7.59	6.71

## 5 Discussion

In this work, we have successfully demonstrated the simultaneous transmission of three different QKD signals multiplexed using OAM fiber modes excited by an integrated photonic chip. Despite the results achieved, multiple improvements should be considered for further deployment of this technology. In particular, improvements on the fabricated silicon photonic chip can be considered. For QKD purposes, for instance, the opto-electronic modules as phase and intensity modulators can be implemented directly on the structure of the chip to form a stand-alone source [24]. In addition, since the current chip structure only supports one polarization, integrating 2-dimensional grating coupler (2DGC) will enable the chip to propagate orthogonal modes, TM and TE, which will enable new applications such as high dimensional quantum communication. Furthermore, to reduce the losses due to the integrated device, more efficient grating couplers with aluminium mirrors could be added [25]. An important factor that impairs the fiber modes extinction ratio is the relative phase of each output port on the chip. In the current configuration, the phase can be controlled and manipulated through thermo-optics modules. Carrier depletion modules, as a substitute to thermo-optics modules, can be investigated to increase the extinction ratio. Indeed, due to the low sensitivity of thermo-optics modules, in our experiment the mode crosstalk increased with the number of active modes, thus increasing the overall QBER and lowering the final secret key rate achievable in the 3-mode test in comparison with 2-mode one. In fact, by exploiting the space division multiplexing (SDM) approach, as demonstrated by D. Bacco and colleagues [26], the expected key rate should follow the simple relation $R_{\text{sk}}^{\text{tot}}=N\;*\;R_{\text{sk}}$ , where *N* is the number of multiplexed channels considered in the system, and *R*_sk_ is the secret key rate. In our proof-of-concept, unfortunately, due to the high mode crosstalk this relation could not be demonstrated. Finally, a better control over the mode crosstalk would also allow us to excite more than three modes simultaneously, thus increasing the overall secret key rate of the protocol.

Furthermore, the relatively high loss of the OAM chip can be attributed to multiple origins. Namely, the two gratings, each contributes 3–4 dB of loss, 3 dB due to ≈1 cm waveguide, and the STAR coupler which simulation and measurements suggest to be responsible for 6–7 dB of loss. Additionally, we observe a slightly mismatch between the chip input spacing and our fiber array. We optimized the coupling such that the output powers at all modes are balanced.

The characterization of the optical modes at the chip output is also a further step to consider. Indeed, the star coupler structure in principle allows for the direct generation of optical modes carrying an OAM. In that case, our integrated device would be regarded as a multiple OAM modes emitter, and it could be implemented in designs for quantum application, for instance as an integrated source of high-dimensional OAM entangled states or for free-space quantum protocols.

The OAM chip can effectively be used as an OAM DEMUX device since it forms a passive element. In principle, the light coupled back to the chip is sampled through the 26 ports. Given that thermo-optical modulators adjusted the relative phase between these ports such that it matches with the phase pattern of the input OAM mode, the light will interfere constructively on the corresponding output in the STAR coupler, translating the chip as an OAM DEMUX device. However, it should be noted that the relatively high loss value renders it currently inefficient specially for QKD purposes. In future the same device could be used for this purpose as well. Furthermore, the 9.15 dB loss due to the optical synchronization signal can be avoided if clock recovery techniques from data such as [27] or a separate channel to share the clock is used. Summarizing, in our work we have excited, through an integrated silicon chip, OAM fiber modes that have been then used to simultaneously multiplex quantum signals. As a concrete application, we have used the OAM multiplexed signals for demonstrating a QKD protocol based on time-bin encoding, showing the applicability of our system. These results are of fundamental importance for further developments of OAM related integrated technologies for quantum communication.
